# Combined serial analysis of gene expression and transcription factor binding site prediction identifies novel-candidate-target genes of *Nr2e1* in neocortex development

**DOI:** 10.1186/s12864-015-1770-3

**Published:** 2015-07-24

**Authors:** Jean-François Schmouth, David Arenillas, Ximena Corso-Díaz, Yuan-Yun Xie, Slavita Bohacec, Kathleen G. Banks, Russell J. Bonaguro, Siaw H. Wong, Steven J. M. Jones, Marco A. Marra, Elizabeth M. Simpson, Wyeth W. Wasserman

**Affiliations:** Centre for Molecular Medicine and Therapeutics at the Child and Family Research Institute, University of British Columbia, 950 West 28th Avenue, Vancouver, BC V5Z 4H4 Canada; Genetics Graduate Program, University of British Columbia, Vancouver, BC V6T 1Z2 Canada; Canada’s Michael Smith Genome Sciences Centre, British Columbia Cancer Agency, Vancouver, BC V5Z 4S6 Canada; Department of Molecular Biology and Biochemistry, Simon Fraser University, Burnaby, BC V5A 1S6 Canada; Department of Medical Genetics, University of British Columbia, Vancouver, BC V6T 1Z3 Canada; Department of Psychiatry, University of British Columbia, Vancouver, BC V6T 2A1 Canada; Current address: Montreal Neurological Institute and Hospital, McGill University, Montréal, QC H3A 2B4 Canada

**Keywords:** SAGE, Nuclear receptor, Nr2e1, Transcriptome, Neocortex, Transcription factor

## Abstract

**Background:**

*Nr2e1* (nuclear receptor subfamily 2, group e, member 1) encodes a transcription factor important in neocortex development. Previous work has shown that nuclear receptors can have hundreds of target genes, and bind more than 300 co-interacting proteins. However, recognition of the critical role of *Nr2e1* in neural stem cells and neocortex development is relatively recent, thus the molecular mechanisms involved for this nuclear receptor are only beginning to be understood. Serial analysis of gene expression (SAGE), has given researchers both qualitative and quantitative information pertaining to biological processes. Thus, in this work, six LongSAGE mouse libraries were generated from laser microdissected tissue samples of dorsal VZ/SVZ (ventricular zone and subventricular zone) from the telencephalon of wild-type (Wt) and *Nr2e1*-null embryos at the critical development ages E13.5, E15.5, and E17.5. We then used a novel approach, implementing multiple computational methods followed by biological validation to further our understanding of *Nr2e1* in neocortex development.

**Results:**

In this work, we have generated a list of 1279 genes that are differentially expressed in response to altered *Nr2e1* expression during *in vivo* neocortex development. We have refined this list to 64 candidate direct-targets of NR2E1. Our data suggested distinct roles for *Nr2e1* during different neocortex developmental stages. Most importantly, our results suggest a possible novel pathway by which *Nr2e1* regulates neurogenesis, which includes *Lhx2* as one of the candidate direct-target genes, and SOX9 as a co-interactor.

**Conclusions:**

In conclusion, we have provided new candidate interacting partners and numerous well-developed testable hypotheses for understanding the pathways by which *Nr2e1* functions to regulate neocortex development.

**Electronic supplementary material:**

The online version of this article (doi:10.1186/s12864-015-1770-3) contains supplementary material, which is available to authorized users.

## Background

The proper development of the mammalian neocortex involves a balance between cell-intrinsic developmental programs and environmental factors. In this process, neurons acting as the backbone of the neuronal circuitry are generated first. These cells arise from the dorsal telencephalon, generating cortical excitatory neurons by radial migration, and the ventral telencephalon giving rise to cortical inhibitory interneurons by tangential migration [1–5]. The neurogenic stage is followed by the integration of glial cells in the circuitry during the gliogenic stage. In mice, neurons are generated from embryonic day 12 (E12) to E18, with astrocytes appearing at around E18 [6, 7]. Ultimately, the neocortex will comprise six different radial layers with cell populations having distinct molecular identities [8].

*Nr2e1* (nuclear receptor subfamily 2, group e, member 1, also known as *Mtll*, *Tlx*, *Tll*, and *tailless*) encodes a transcription factor important in the process of neocortex development [9, 10]. This complex cellular process involves a careful balance between proliferation of neural stem cells (NSC), and the proper temporal differentiation of progenitor cells (PC) (i.e. neurons versus glia). *Nr2e1* is expressed along the ventricular zone (VZ) of the dorsal telencephalon during neocortex development and is crucial for NSC self-renewal and maintenance [11–14]. Absence of *Nr2e1* in mouse embryos reduces the number of PC populating the VZ and subventricular zone (SVZ) during development, which results in reduced thickness of the cortical plate [9]. The reduction in PC populating the VZ is more prominent in the caudal telencephalon whereas the reduction in the SVZ is seen at all rostrocaudal levels during development. This cell-reduction ultimately results in defects in structures generated later, such as the upper cortical layers (layers II and III), the dentate gyrus, and the olfactory bulb [9, 10]. Absence of *Nr2e1* in mouse embryos also results in premature neurogenesis, which contributes to the defects in the upper cortical layers [9].

Previous work has shown that a nuclear receptor transcription factor can have hundreds of target genes [15], and the most extensively studied nuclear receptors are estimated to bind more than 300 co-interacting proteins [16, 17]. However, recognition of the critical role of nuclear receptor *Nr2e1* in NSC and neocortex development is relatively recent [11, 12, 18–20], thus the molecular mechanisms involved for this nuclear receptor are only beginning to be understood. First, in forebrain development, *Nr2e1* has been shown to regulate cell cycle progression via its interaction with the tumour suppressor gene *Pten*, and the cyclin-dependent kinase inhibitor *p21* [11]. This involves a repressive mechanism mediated via the interaction of Nr2e1 with chromatin modifier proteins such as members of the histone deacetylase family (HDACs), and the demethylase protein LSD1 (KDM1A) [14, 21]. Second, the balance between NSC proliferation and differentiation has been demonstrated to be under the control of regulatory loops involving both *Nr2e1*, and microRNA encoding genes such as *mir-9*, *miR-137*, and *let-7d* [22–24]. This phenomenon includes an intricate network formed by the ability of *let-7d* and *mir-9* to silence *Nr2e1* expression by binding the 3′ UTR regions of this gene and the ability of *Nr2e1* to inactivate the expression of *mir-9* in a first feedback loop [22, 24]. A second loop has been reported that includes the repression of the co-interactor *Lsd1* by *miR-137* that can be relieved by the repression of *miR-137* by *Nr2e1* [23]. Finally, *Nr2e1* has been shown to act as a transcriptional activator of the deacetylase gene *Sirt1*, which has a role in promoting neuronal differentiation [25, 26]. Thus, we hypothesize there is still much to learn about the molecular mechanisms underlying the role of NR2E1 in NSC and neocortex development. Hence, we have undertaken additional research on these mechanisms, especially focused on *in vivo* analyses, to inform our understanding of neocortex development.

Large-scale transcriptome-profiling experiments, using methodologies such as serial analysis of gene expression (SAGE), have given researchers the advantages of both qualitative and quantitative information pertaining to biological processes. SAGE analysis relies on sequencing and quantification of short (14 bp) cDNA fragments called tags, which are derived from messenger RNA transcripts [27]. This approach is considered an open transcriptome technology as no *a priori* knowledge of the transcript sequences is required [28]. For the mammalian central nervous system, SAGE profiling experiments have been used to generate knowledge on a variety of topics including; fundamental studies on brain development [29], connectivity, and aging [30–32], as well as specific neuropathologies and drug responses [33–36]. Advancement in SAGE library generation such as SAGE-lite [37], which enabled the use of extremely small quantities of tissues such as those from laser capture microdissection (LCM), and LongSAGE, which improved tag-to-gene mapping by generating longer tag fragments (21 bp) [38], made these approaches particularly appropriate to reveal *in vivo* molecular changes in neocortex development. One of the inherent challenges in transcriptome profiling is the effective analysis of large-scale datasets to optimize extraction of relevant biological meaning. By producing LongSAGE libraries at multiple developmental times, in the presence and absence of *Nr2e1*, we generated a rich dataset for comparative analysis. Additionally, we took advantage of the intrinsic nature of transcription factors, which regulate gene expression by binding to specific DNA sequences, and used it to further hone our gene list. This was partly based on the power of transcription-factor-discovery-motif algorithms that, when coupled to cross-species genome comparisons or phylogenetic footprinting, have proven successful in making reliable binding site predictions [39–42]. Returning to biology to further validate the bioinformatic studies, we of course used the literature, but most importantly, we also tested our primary new hypothesis *in vitro* by embryonic stem cells (ESC) differentiation and *in vivo* during brain development. Thus, in this work, we used a novel approach, implementing multiple computational methods to generate significant-novel-biological information regarding the molecular mechanisms underlying the role of nuclear receptor *Nr2e1* in neocortex development.

## Results and discussion

### LongSAGE libraries generated from laser capture microdissection tissues

**Fig. 1:**
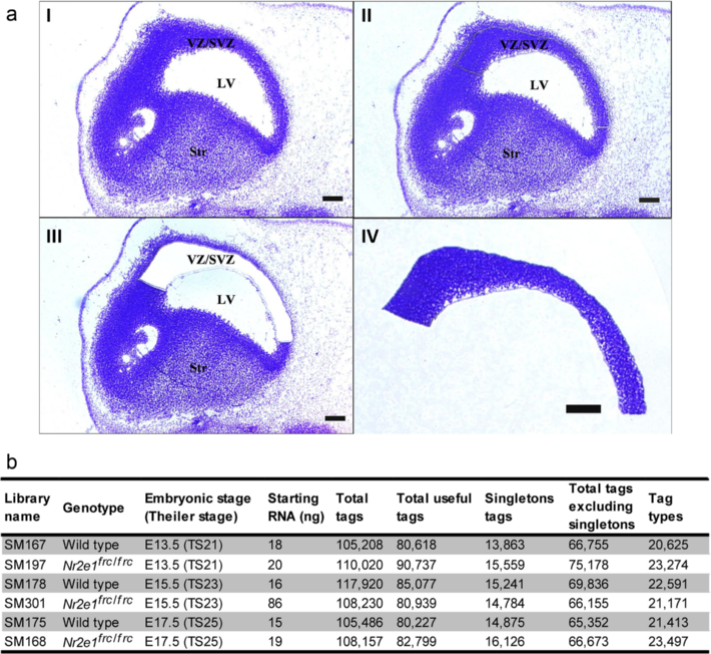
Laser microdissected LongSAGE libraries were used to reveal the transcriptome of Wt and *Nr2e1* ^*frc*/*frc*^ embryos. **a** The laser capture microdissection (LCM) procedure. ((**a**), I-IV) Embryonic day 13.5 sagittal sections stained with cresyl violet. ((**a**), II) The ventricular/subventricular zone (VZ/SVZ) of the dorsal lateral telencephalon cut with laser. ((**a**), III) The VZ/SVZ removed by LCM. ((**a**), IV) The VZ/SVZ of the dorsal lateral telencephalon captured by LCM for RNA extraction. LV, Lateral Ventricle; Str, striatum. Scale bars, 100 μm. **b** The composition of LongSAGE libraries. Column one presents the name of the library; columns two and three, the genotype and developmental stage respectively; column four, the amount of RNA used as starting material; and columns five to nine, the number of tags for each library depending on the filtering criteria used

To identify novel-candidate-target and co-interacting genes for the nuclear receptor *Nr2e1*, we favoured an *in vivo* source of RNA in order to most accurately capture molecular events occurring during neocortex development. Thus, LongSAGE libraries were prepared using RNA purified from tissues obtained by LCM of the VZ/SVZ, of the dorsal-lateral telencephalon, from Wt and *Nr2e1*^*frc*/*frc*^ embryos. This work was undertaken at three different developmental time points (E13.5, E15.5, and E17.5), which are known to express *Nr2e1* in the dissected region [9, 11, 18, 43] (Fig. 1a). These libraries were sequenced to a depth ≥100,000 tags (total number of tags per libraries, see Fig. 1b). To generate tags for analysis, we used a filtering procedure involving the DiscoverySpace 4.0 application (http://www.bcgsc.ca/platform/bioinfo/software/ds) (filtering details, see Methods) [44]. On average, ~24 % of the total tags per library were discarded in this procedure resulting in a useful tag population averaging ~83,000 tags per library, and corresponding to ~25,000 tag types per library (Fig. 1b). Singleton tags (tags counted only once) constituted ~18 % of the useful tags population per library and ~68 % of the tag type population per library (Fig. 1b). These numbers were consistent with previously published results, obtained using a similar filtering procedure [45].

### LongSAGE libraries differential statistical analyses and gene IDs recovery

**Fig. 2:**
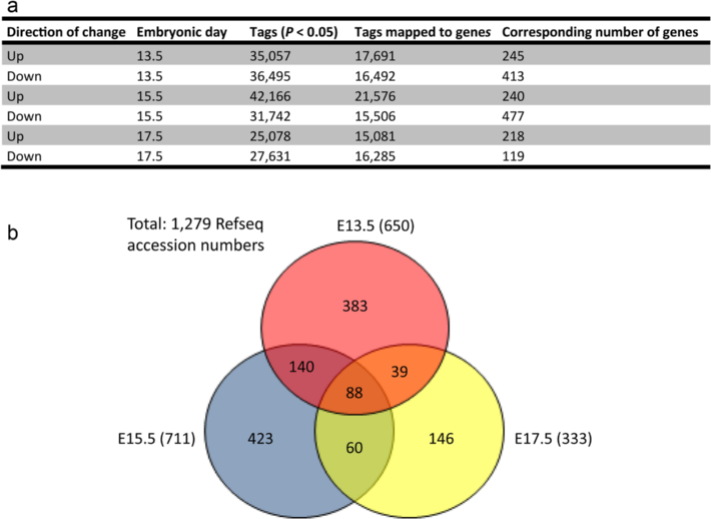
Comparative transcriptome investigation of Wt and *Nr2e1* ^*frc*/*frc*^ embryos yielded hundreds of candidate-differentially-regulated genes. **a** Details of the number of differentially abundant tags (increased or decreased) and the corresponding number of genes between Wt and *Nr2e1* ^*frc*/*frc*^ embryos at each time point. In the exceptional case of a gene having both a significantly up and down tag, it was counted in both categories. Column one presents the direction of change; column two, the embryonic day of tissue harvest; column three, the number of tags that had significantly different counts between Wt and *Nr2e1* ^*frc*/*frc*^ embryos; column four, the number of tags having significant different counts that mapped to genes found in the Ensembl (v66) and Refseq (v52) gene collections; and column five, the number of Refseq genes mapped by the corresponding tags. **b** The Venn diagram presents the number of up- and down-regulated genes that were exclusive or shared at each embryonic day (E13.5, E15.5, and E17.5). Bracketed numbers correspond to the total of differentially-regulated genes between Wt and *Nr2e1* ^*frc*/*frc*^ embryos at the corresponding time point

The Audic-Claverie significance test, implemented in the DiscoverySpace 4.0 application, was used to perform statistical analyses on the filtered tags [44, 46]. Tags differentially expressed between Wt and *Nr2e1*^*frc*/*frc*^libraries at each time point (E13.5, E15.5, and E17.5), and falling within the confidence interval of 95 % (*P* < 0.05), according to the Audic-Claverie significance test, were retained for further analyses. The results for tags significantly increased or decreased in abundance at each time point are shown in Fig. 2a. The proportion of differentially abundant tags (either increased or decreased) varied between 15 to 25 % when compared to the combined numbers of useful tags found in the Wt and *Nr2e1*^*frc*/*frc*^ library at each time point (e.g. (Up at E13.5 “Tags (*P* < 0.05)” (Fig. 2a)/(Wt + *Nr2e1*^*frc/frc*^ at E13.5 “Total useful tags” (Fig. 1b))) × 100). LongSAGE tags were mapped to RefSeq (v52) and Ensembl (v66) databases [45]. On average, 52 % of the differentially abundant tags mapped to genes (average of (“Tags mapped to genes”/“Tags (*P* < 0.05)”)×100 for each library, Fig. 2a). The number of Refseq accession IDs corresponding to differentially abundant tags at the three different time points are also shown in Fig. 2a. These accession numbers, corresponding to Refseq genes, were retrieved and used in the subsequent analyses.

We next looked at the genes that were differentially regulated between the Wt and *Nr2e1*^*frc*/*frc*^ libraries at the E13.5, E15.5, and E17.5 time points. This resulted in a total of 1279 Refseq accession numbers, originating from a corresponding list of 1387 tag sequences (Additional file 1: Table S1 and Additional file 2: Table S2), and distributed according to the Venn diagram in Fig. 2b. Interestingly, when performing the analyses, on average 6 genes per time point corresponded to tags that were found in both the up and down regulated populations (data not shown). This suggested that the tags mapped to these genes were corresponding to alternative transcripts that were expressed in opposing directions when comparing Wt and *Nr2e1*^*frc*/*frc*^ libraries. The Venn diagram results also demonstrated that on average 54 % of the differentially-regulated genes (combined up and down) were specific for each time point: E13.5, 59 % ((383/650) × 100); E15.5, 59 % ((423/711) × 100); and E17.5, 44 % ((146/333) × 100). Furthermore, on average 17 % of the genes overlapped between at least two time points: E13.5 and E15.5, 20 % (((140 + 88)/(650 + 423 + 60)) × 100); E15.5 and E17.5, 17 % (((88 + 60)/(711 + 146 + 39)) × 100); and E13.5 and E17.5, 15 % (((88 + 39)/(650 + 146 + 60)) × 100). Finally, only 6.9 % of the genes overlapped between the three time points ((88/1279) × 100).

### LongSAGE expression results suggested distinct roles for *Nr2e1* in different stages of neocortex development

**Fig. 3:**
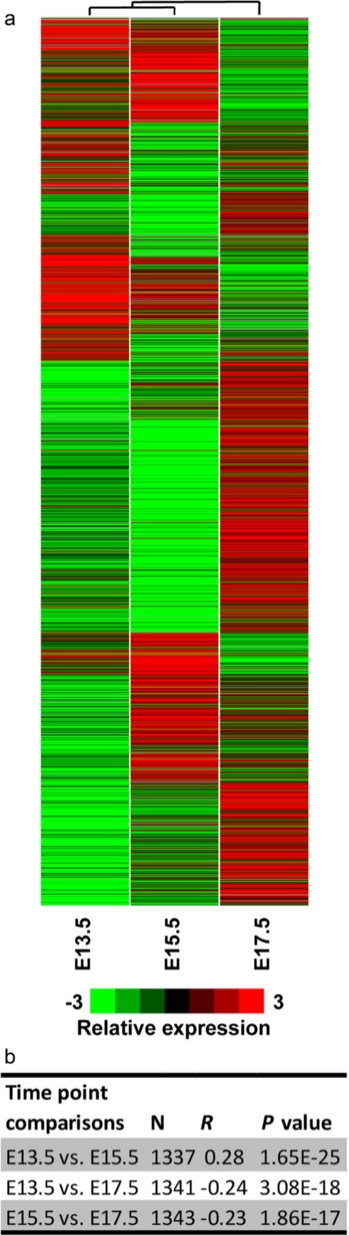
Hierarchical clustering suggested a stage-specific role for *Nr2e1* in neocortex development. **a** Tag numbers for each of the corresponding 1279 genes were retrieved from DiscoverySpace and hierarchical-clustering was performed prior to visualization in a heat-map; displaying significant-differential-tag ratios from Wt vs. *Nr2e1* ^*frc*/*frc*^ libraries. The relative expression was calculated based on the tag ratios from Wt vs. *Nr2e1* ^*frc*/*frc*^ libraries and corrected to account for library sizes; (observed tag counts/total useful tags) X 100,000. Tags having a count value of “0” (no expressed tags) were adjusted to a value of “1” for fold change calculations only. Green, down; red, up; black, no difference; grey, no expressed tags. **b** Embryonic-stage-specific differences in expression profiles of the 1279 differently abundant genes in neocortex development of Wt vs. *Nr2e1* ^*frc*/*frc*^ embryos was demonstrated using Spearman rank correlation. Spearman rank correlation was performed on gene lists from pairs of embryonic stages using STATISTICA. Results revealed a significant positive correlation between the lists corresponding to early and mid-neurogenic stages (E13.5 vs. E15.5); whereas significant negative correlations were obtained with the tag ratios corresponding to the early and mid-neurogenic stages versus the early gliogenic stages (E13.5 vs. E17.5, and E15.5 vs. E17.5). Column one presents the time-point comparisons; column two, the valid number of genes included in the analysis; columns three and four, the corresponding *R* and *P* values obtained by Spearman rank correlation

To understand the role of *Nr2e1* in gene expression during neocortex development, we performed hierarchical clustering on the tag ratio values corresponding to each of the 1279 Refseq accession numbers of differentially-regulated genes (Fig. 3, Additional file 1: Table S1 and Additional file 2: Table S2). Tag sequences and corresponding tag counts of the 1279 Refseq accession numbers were retrieved for each LongSAGE library using the DiscoverySpace 4.0 application. Fold changes from tags statistically differentially abundant, at least at one time point between the Wt and *Nr2e1*^*frc*/*frc*^ libraries, were calculated as previously described [45], and hierarchical clustering was performed using the Gene Cluster software as described in Methods [47]. The clustering results were visualized in a heat-map display using Java TreeView (Fig. 3a) [48]. Spearman-rank-ordering correlation was additionally performed on the fold changes dataset at each time point as described in Methods. The results demonstrated that at the E13.5 and E15.5 time-points, differential tag ratios correlated positively (Spearman *R =* 0.28, *P* < 0.001). In contrast, comparing E13.5 and E17.5, as well as E15.5 and E17.5 yielded negative correlation values (E13.5 vs. E17.5, Spearman *R = −*0.24, *P* < 0.001; E15.5 vs. E17.5, Spearman *R = −*0.23, *P* < 0.001) (Fig. 3b). This demonstrated that the differential-tag ratio found between the E13.5, and E15.5 libraries were more similar than the one observed in the E17.5 library. This also suggested that *Nr2e1* expression has a more similar effect on genes in early and mid-stages of neurogenesis, than during the switch from neurogenesis to gliogenesis occurring around E17.5. These results correlated with previously published observations, demonstrating a progression of the *Nr2e1*-null phenotype during neocortex development, with a greater effect between E13 and E15 [9].

### Bioinformatics analyses for the prediction of *Nr2e1* direct targets

**Fig. 4:**
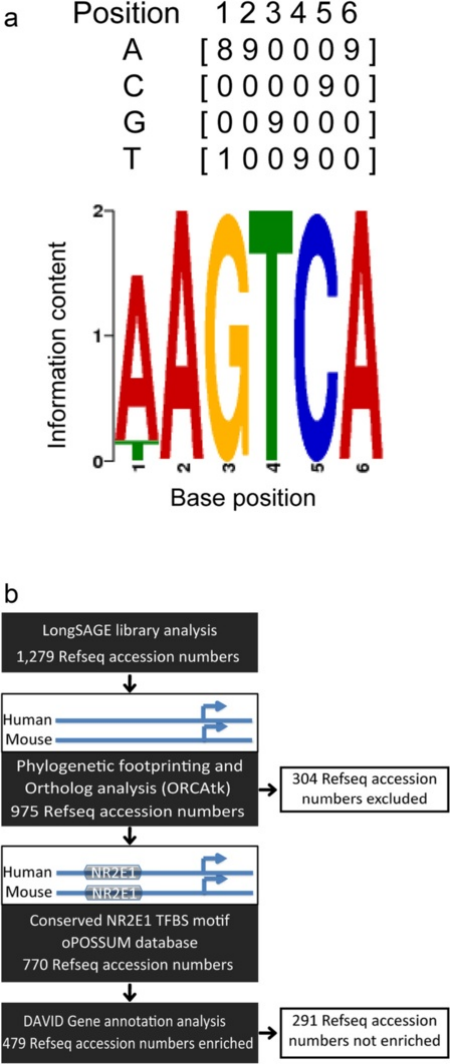
Novel implementation of three computational methods to generate a focused list of biologically-relevant *Nr2e1-*candidate-target genes. **a** Data mining from the literature allowed generation of a position-weight matrix representing the binding properties of NR2E1. The matrix and resulting logo are presented. **b** Flow chart describing the novel implementation of three computational methods to generate a focused list of biologically-relevant *Nr2e1-*candidate-target genes. DiscoverySpace was used to generate a compiled list of 1279 Refseq accession numbers, corresponding to genes differentially regulated between Wt and *Nr2e1* ^*frc*/*frc*^ embryos. A customized oPOSSUM database of predicted conserved TFBS was created by first aligning all orthologous human-mouse genes using ORCAtk. During this alignment, 304 of the 1279 differentially regulated genes were excluded due to a lack of ortholog information or poor ORCAtk alignment quality. Then the remaining 975 genes with conserved promoter regions were scanned with the NR2E1 matrix (as well as all the vertebrate matrices from the JASPAR CORE collection of transcription factor binding site profiles). Of the 975 scanned genes, 770 contained conserved NR2E1 binding sites. These 770 Refseq accession numbers were submitted to a gene ontology (GO) term analysis using DAVID. From the DAVID analysis, 479 Refseq accession numbers were found to be enriched in GO term categories related to biological processes

Considering that *Nr2e1* encodes for a transcription factor, and that transcription factors regulate transcription by binding the promoter regions of their target genes; we hypothesized that a list of genes, differentially regulated between Wt and *Nr2e1*^*frc/frc*^, would comprise genes containing Nr2e1 binding sites within their promoter regions. Thus, interrogation of our pooled list of 1279 Refseq accession numbers was undertaken using three different software tools; the ORCA toolkit (tk) to perform the initial orthologous-sequence alignment and phylogenetic footprinting [49], a customized version of oPOSSUM for prediction and storage of transcription factor binding sites (TFBSs) (http://www.cisreg.ca/oPOSSUM/) [39, 40], and a DAVID GO term analysis to evaluate if the resulting genes were found in biological processes relevant to *Nr2e1* (http://david.abcc.ncifcrf.gov/summary.jsp) [50, 51]. The “modified” version of the oPOSSUM database used a position-weight matrix (PWM) that we designed based on the nine sequences available from the literature, which were known to be bound by NR2E1 (Additional file 3: Table S3) [21, 22, 52–56]. The resulting matrix and logo are depicted in Fig. 4a.

The results from these sequential analyses are summarized in the flowchart of Fig. 4b. ORCAtk orthologous sequence alignments between human and mouse for each gene was initially performed; resulting in the exclusion of 304 Refseq accession numbers due to poor conservation between human and mouse within the promoter sequences of these genes. This resulted in 975 Refseq accession numbers that were used in the modified oPOSSUM database. Of these 975 accession numbers, 770 (79 %) were found to have predicted binding sites for NR2E1 within their promoter regions (Fig. 4b) (Additional file 2: Table S2). [57, 58] These 770 accession numbers were further studied in a GO term enrichment analysis using the DAVID service. The 770 Refseq accession numbers were first converted to DAVID IDs using the DAVID knowledgebase, and then compared to the DAVID mouse-background list of genes [50, 59]. The enrichment results were visualized using the functional annotation module based on the relevance for each enriched gene to “biological process” with an initial *P* value < 0.1, using the modified Fisher exact test (EASE score) [51, 60, 61]. In this process, 291 Refseq accession numbers were discarded as they were not enriched in our list compared to the mouse background (Fig. 4b). The remaining 479 Refseq accession numbers were interrogated based on their “biological process” terms. Only terms with a *P* value < 0.05 after multiple test correction, using the Bonferroni approach [59, 62], were considered interesting for further investigation. Table 1 shows the list of GO terms passing this criterion, Additional file 2: Table S2 list the differentially expressed genes within these GO terms. As expected, numerous terms related to cell cycle regulation were found after performing the GO term enrichment analysis on the 770 Refseq list. However, terms related to cell cycle regulation were also found in a similar analysis, using the initial 1279 Refseq list. This suggested that genes involved in cell cycle regulation were differentially expressed in our LongSAGE results when comparing Wt to *Nr2e1*^*frc*/*frc*^, but were not enriched for the presence of NR2E1 binding sites within the promoter regions. In contrast, the term “nervous system development” (*P* < 0.01), with 64 differentially-regulated genes, was found to be enriched only after performing the analysis on the 770 Refseq list, again, suggesting the presence of NR2E1 binding sites within the promoter regions of genes enriched for this term. Interestingly, similar results were also obtained using a different GO term enrichment software; “GOstats” yielded significant results for the “nervous system development” term (*P* < 0.001) [63]. Thus, we subsequently used the “nervous system development” gene list from the “DAVID analysis” for further investigations.

**Table 1:** Gene ontology (GO) term analysis revealed enrichment in relevant biological processes

GO identifiers and terms	No. of differentially expressed genes^a^	*P* value after Bonferroni^b^
GO:0006396 ~ RNA processing	55	4.39E-10
GO:0016070 ~ RNA metabolic process	68	7.54E-09
GO:0044267 ~ cellular protein metabolic process	132	2.96E-04
GO:0007067 ~ mitosis	26	3.12E-04
GO:0000280 ~ nuclear division	26	3.12E-04
GO:0015031 ~ protein transport	56	3.58E-04
GO:0045184 ~ establishment of protein localization	56	4.56E-04
GO:0000087 ~ M phase of mitotic cell cycle	26	4.66E-04
GO:0046907 ~ intracellular transport	42	6.08E-04
GO:0030163 ~ protein catabolic process	49	1.13E-03
GO:0044265 ~ cellular macromolecule catabolic process	52	1.27E-03
GO:0000279 ~ M phase	31	2.35E-03
GO:0051246 ~ regulation of protein metabolic process	35	3.82E-03
GO:0007399 ~ nervous system development^c^	64	5.13E-03
GO:0022403 ~ cell cycle phase	33	6.36E-03
GO:0019538 ~ protein metabolic process	151	6.91E-03
GO:0006886 ~ intracellular protein transport	28	3.18E-02
GO:0034613 ~ cellular protein localization	29	4.95E-02

^a^352 total non-overlapping differentially expressed genes

^b^ http://david.abcc.ncifcrf.gov/ease/Help/Technical%20details/Overrepresentation%20analysis.htm

^c^term used in subsequent analyses

### Differential expression results validated by literature

**Fig. 5:**
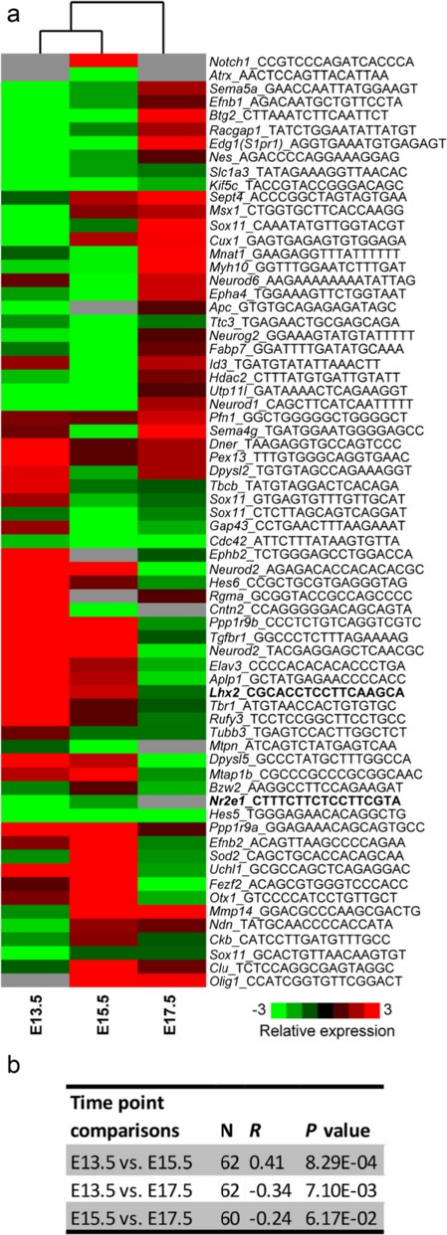
Hierarchical clustering suggested a stage-specific role for *Nr2e1* in nervous system development. **a** Tag numbers for each of the 64 genes were retrieved from DiscoverySpace and hierarchical-clustering was performed prior to visualization in a heat-map; displaying significant-differential-tag ratios from Wt vs. *Nr2e1* ^*frc*/*frc*^ libraries. The relative expression was calculated based on the tag ratios from Wt vs. *Nr2e1* ^*frc*/*frc*^ libraries and corrected to account for library sizes; (observed tag counts/total useful tags) X 100,000. Tags having a count value of “0” (no expressed tags) were adjusted to a value of “1” for fold change calculations only. Green, down; red, up; black, no difference; grey, no expressed tags; bold, key genes in this analysis. **b** Embryonic-stage-specific differences in expression profiles of “nervous system development” genes in neocortex development of Wt vs. *Nr2e1* ^*frc*/*frc*^ embryos was demonstrated using Spearman rank correlation. Spearman rank correlation was performed on gene lists from pairs of embryonic stages using STATISTICA. Results revealed a significant positive correlation between the lists corresponding to early and mid-neurogenic stages (E13.5 vs. E15.5); whereas a significant negative correlation was obtained with the tag ratios corresponding to the early neurogenic and early gliogenic stages (E13.5 vs. E17.5). No significance was obtained between the mid-neurogenic and early gliogenic stages (E15.5 vs. E17.5). Column one presents the time-point comparisons; column two, the valid number of genes included in the analysis; columns three and four, the corresponding *R* and *P* values obtained by Spearman rank correlation

We used the tag ratio values of the 64 differentially expressed genes found in the “nervous system development” GO term category to perform hierarchical clustering (Fig. 5a, Additional file 2: Table S2). We used the same hierarchical procedure as the one described for the 1279 genes list. Similarly to previously obtained results, the E13.5 and E15.5 time-points, differential tag ratios correlated positively (Spearman *R =* 0.41, *P* < 0.001), and the E13.5 and E17.5 yielded a negative correlation value (Spearman *R = −*0.34, *P* < 0.001). However, no significance was observed when comparing the E15.5 and E17.5 time points (Fig. 5b). This suggested that the differential-tag ratio found between the E13.5, and E15.5 libraries were more similar than the one observed in the E17.5 library; highlighting again the possibility of distinct roles for *Nr2e1* in the neurogenic versus early gliogenic stages of neocortex development.

As expected, all the *Nr2e1*^*frc*/*frc*^ libraries showed no tags for *Nr2e1*. Interestingly, even the Wt libraries, despite being obtained by LCM for a focused region of *Nr2e1* expression, showed low abundance of *Nr2e1* tags (E13.5, 4; E15.5, 2; and E17.5, 0). Thus, only E13.5 reached significant differential expression between Wt and *Nr2e1*^*frc*/*frc*^ (−4.5 fold, *P* < 0.05). As expected, the number of tags mapping to *Nr2e1* in the Wt libraries showed a declining trend (Wt E13.5 vs. Wt E17.5, *P* = 0.06). This is in agreement with published and publicly-available expression results [18] (Allen Mouse Brain Atlas, http://www.brain-map.org/); where *Nr2e1* expression has been observed as early as E8, peaks at E13, sharply decreases until E18, and is barely detectable in new-born brains [18]. Hence, at the time point of lowest *Nr2e1* expression (E17.5) the LongSAGE approach was insufficiently sensitive to detect this latter gene transcript. Interestingly, our bioinformatics enrichment analysis included *Nr2e1* in the list of genes with predicted NR2E1 binding sites within their promoter regions, adding support to previous observations proposing a self-regulating mechanism for *Nr2e1* [22, 64].

When analysing large-scale transcriptome-profiling datasets, the overall level of expression is an important factor influencing the outcome of statistical significance. In our LongSAGE libraries, *Pten* and *P21* (*Cdkn1a*), two direct targets of *Nr2e1* [11, 14, 21], were expressed at low levels in the VZ/SVZ (total number of tags, *Pten*: E13.5, Wt 1, *Nr2e1*^*frc*/*frc*^ 5; E15.5, Wt 1, *Nr2e1*^*frc*/*frc*^ 1; and E17.5, Wt 0, *Nr2e1*^*frc*/*frc*^ 1; *P21*: E13.5, Wt 0, *Nr2e1*^*frc*/*frc*^ 0; E15.5, Wt 1, *Nr2e1*^*frc*/*frc*^ 3; and E17.5, Wt 1, *Nr2e1*^*frc*/*frc*^ 2) and thus did not reach significance in terms of differential expression between Wt vs. *Nr2e1*^*frc*/*frc*^ libraries. In contrast, *Nestin*, a common marker of proliferating neural progenitors, which was expressed at mid to high levels, was significantly down regulated in *Nr2e1*^*frc*/*frc*^ at E13.5 when compared to Wt (−7.3 fold, *P* < 0.05) (Fig. 5a, Additional file 1: Table S1). This correlated with the previously published observation of reduced numbers of *Nestin-*positive cells in the VZ of *Nr2e1*-null embryos at E14.5 [11]. In addition, our data suggests that the mechanism involves a direct-up regulation by *Nr2e1* in Wt*,* as *Nestin* was found within the bioinformatics enrichment analysis genes with predicted NR2E1 binding sites. Another example of our expression results being supported by the literature is the basic helix-loop-helix (bHLH) gene *Neurog2*, which was significantly down regulated in *Nr2e1*^*frc*/*frc*^ embryos at both E13.5 and E15.5 when compared to Wt (E13.5, −2.8 fold, *P* < 0.001; E15.5, −5.5 fold, *P* < 0.001) (Fig. 5a, Additional file 1: Table S1). These results correlated with the previously published observations of reduced expression of *Neurog2* in double mutants embryos for *Pax6* and *Nr2e1* in the rostral granular zone during neocortex development [65]. Disruptions in *Neurog2* expression are also characteristic of alterations in the pallio-subpallial boundary observed in *Nr2e1*-null embryos [66]. Additionally, downstream candidate genes of the pathway regulated by *Neurog2* (i.e. *Neurod2*, and *Tbr1*) were found differentially expressed in our LongSAGE comparison analysis, arguing in favour of a direct role for *Nr2e1* in regulating this specific pathway during neocortex development (Fig. 5a, Additional file 1: Table S1) [65].

### TFBS overrepresentation analysis revealed novel-candidate-NR2E1 co-interactors

Spatial-temporal gene expression is, in general, regulated by the dual ability of transcription factors to bind specific DNA sequences and to form complexes with other regulatory proteins. NR2E1 has previously been shown to mediate gene regulation with co-interacting partners; forming regulatory complexes that lead to either direct-target-gene repression or activation [14, 21, 25, 26]. Interestingly, nuclear receptors have also been shown to mediate gene regulation via interaction with other transcription factors as co regulators [64, 67, 68]. Based on our Spearman rank ordering results, we hypothesized that the striking difference in direction of correlation for differentially abundant tags between the E13.5-E15.5, E13.5-E17.5, and E15.5-E17.5 time points, was largely due to the presence of different Nr2e1 interacting partners at different times in development. To discover novel candidate co-interactors of Nr2e1, we designed a computational experiment to identify TFBS within the vicinity of the predicted NR2E1 binding sites for each differentially-regulated gene found in the GO term category “nervous system development”. The identified binding sites were then scored for their enrichment compared to a randomized list of genes, thereby generating both a Z and Fisher score. Potential TFBSs having a Z-score value > 10 and a Fisher score value < 0.01 were considered enriched and kept for further characterization as candidate-NR2E1 co-interactors (Table 2). We further ascertained the significance of our candidate-NR2E1 co-interactors list by performing analyses on random sets of 64 genes extracted from the initial list of 770 genes obtained through the oPOSSUM-NR2E1 binding motif interrogation step. Corresponding empirical *P* values based on the Z-scores and Fisher scores of each of the candidate-NR2E1 co-interactors were extracted from the random sets of 64 genes and are summarized in Additional file 4: Table S4.

**Table 2:** SOX9 revealed as a candidate co-interactor of Nr2e1 for genes of the “nervous system development”

Transcription factors	No. of background hits	No. of background non-hits	No. of target hits	No. of target non-hits	Z score	Fisher score	ABA Expression at E13.5	ABA Expression at E15.5	ABA Expression at E18.5	Other expression resources	Expression pattern score
SP1	190	310	41	23	20.40	6.53E-05	Not available	Not available	Not available	Ubiquitous^a^	++
Nobox	248	252	47	17	17.70	2.13E-04	Weak, Ubiquitous	Moderate, Ubiquitous	Weak	Not applicable	+
**SOX9**	**172**	**328**	**40**	**24**	**16.97**	**1.58E-05**	**Moderate, VZ/SVZ**	**Moderate, VZ/SVZ**	**Strong, VZ/SVZ**	**Not applicable**	**+++**
Arnt-Ahr	243	257	48	16	15.76	4.38E-05	Weak, Ubiquitous	Moderate, Ubiquitous	Moderate, Ubiquitous	Not applicable	++
Nkx2-5	309	191	58	6	15.12	7.92E-07	Not available	Not available	Not available	Absent^b^	-
Gfi1	199	301	42	22	14.27	7.74E-05	Not available	Not available	Not available	Strong, ubiquitous^c^	++
Lhx3	74	426	20	44	14.00	1.58E-03	Weak	Weak	Weak	Not applicable	+
TAL1-TCF3	93	407	26	38	13.60	1.22E-04	Moderate, Ubiquitous	Moderate, Ubiquitous	Weak, Ubiquitous	Not applicable	+
NHLH1	61	439	22	42	13.57	2.00E-05	Strong, Neocortex	Weak, Neocortex	Weak	Not applicable	++
Myb	211	289	46	18	13.49	5.86E-06	Weak, Neocortex	Weak, Neocortex	Weak	Not applicable	++
Roaz (Zfp423)	59	441	19	45	12.99	3.31E-04	Not available^a^	Not available^a^	Not available	Strong, ubiquitous^d^	++
FOXI1	189	311	36	28	12.27	3.72E-03	Weak	Weak	Weak	Not applicable	+
Prrx2	296	204	53	11	12.26	1.13E-04	Weak	Weak	Weak	Not applicable	+
Cebpa	131	369	37	27	11.92	6.32E-07	Weak	Weak	Weak, Neocortex	Not applicable	+
Foxa2	177	323	35	29	11.38	2.38E-03	Weak, Ubiquitous	Weak, Ubiquitous	Weak, Ubiquitous	Not applicable	+
NFYA	68	432	18	46	11.36	3.60E-03	Moderate, Ubiquitous	Moderate, Ubiquitous	Moderate, Ubiquitous, Neocortex	Not applicable	++
Sox17	237	263	48	16	11.06	2.06E-05	Weak	Weak	Weak	Not applicable	+
Sox5	269	231	51	13	10.61	4.18E-05	Strong, Neocortex	Strong, Neocortex	Strong, Neocortex	Not applicable	++
SRY	267	233	48	16	10.11	6.56E-04	Weak	Weak	Weak	Not applicable	+
MYC-MAX	32	468	12	52	10.07	1.87E-03	Moderate, Ubiquitous	Moderate, Ubiquitous	Moderate, Ubiquitous, Neocortex	Not applicable	++

Bold, most relevant transcription factor according to both the statistical and expression pattern scores; Not available, no expression data available in the ABA (http://www.brain-map.org/, accessed January 10^th^ 2014); Not applicable, data available in ABA so no need to access other resources

^a^Gray et al. (PMID: 15618518) showed expression in the central nervous system, including the brain ventricular layers at E13.5

^b^Expression restricted to the developing striatum at E14.5 according to Eurexpress (http://www.eurexpress.org/)

^c^Wallis et al. (PMID: 12441305) showed expression in the developing forebrain at E12.5

^d^Expression strong and ubiquitous in the developing brain according to Eurexpress and GenePaint at E14.5 (http://www.genepaint.org/Frameset.html)

The relevance of these enriched TFBS was also evaluated based on the expression pattern of their corresponding transcription factors. For this, we primarily used data from the Allen Mouse Brain Atlas (http://www.brain-map.org/) at three different time points (E13.5, E15.5, and E18.5), and included data from other publically-available resources as required (Table 2) [69].

**Fig. 6:**
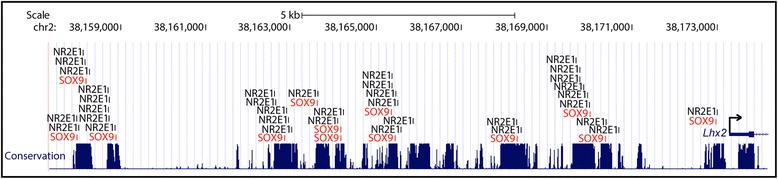
*Lhx2* contains enriched clusters of NR2E1 and SOX9 binding sites in highly conserved regions. Presented is the ~17 kb of genomic sequence 5′ to, and surrounding, the *Lhx2* transcription start site. NR2E1 binding sites are indicated in black, and SOX9 binding sites are indicated in red. NR2E1 binding site coordinates were derived from the position-weight matrix (PWM) described in the current manuscript; SOX9 binding sites coordinates were derived from the PWM stored in the JASPAR database (http://jaspar.binf.ku.dk/cgi-bin/jaspar_db.pl). Conservation, alignment of 100 vertebrate species from the University of California, Santa Cruz genome browser (UCSC: http://genome.ucsc.edu/); thick horizontal line, first exon of *Lhx2*; arrow, transcription start site and sense

The expression data and statistical scores obtained most strongly supported the biological relevance of SOX9 (Z-score: 16.97, empirical *P* value: < 0.001; Fisher score 1.58E-05, empirical *P* value: = 0.001); a member of the SRY-box family. Examination of our LongSAGE data revealed the presence of tags corresponding to *Sox9* throughout the three different time points for both genotypes (data not shown). Additionally*, Sox9* has been reported to function in neural-stem/progenitor-cell regulation; as does *Nr2e1*. Together these data support the hypothesis that SOX9 acts as a co-interactor of NR2E1 [70]. Thus, the differentially-regulated genes found in the GO term category “nervous system development”, and the number of predicted TFBSs for both NR2E1 and SOX9 in the promoter region of these genes, are presented in Table 3 (Additional file 2: Table S2). These 40 genes represent a rich resource for the biological examination of *Nr2e1* downstream targets. Here we pursue the top candidate *Lhx2*, a LIM-homeobox transcription factor. *Lhx2* had the highest number of predicted binding sites for both NR2E1 and SOX9; 35 and 13 respectively. Visualization of the predicted binding sites within the promoter region of *Lhx2* revealed a clustered distribution that was located within highly conserved DNA (Fig. 6). Localization within conserved DNA further suggested a function for these binding sites throughout evolution. Evidence from the literature highlights a spatial-temporal dynamic role for *Lhx2* in the developing forebrain. Early in development (E10.5-E11.5), *Lhx2* has been shown to work as a fate determinant of cortical identity [71]. Later in development, distinct roles have been described for *Lhx2* depending on the forebrain structures involved; including a role in regulating progenitor cell differentiation in neocortex development (E11.5-E13.5) and a role in the neurogenic to gliogenic switch in hippocampal development (E14.5-E15.5) [72, 73]. Thus, our data, combined with the literature, support *Lhx2* as a direct target of co-regulation by NR2E1 and SOX9.

**Table 3:** Overrepresentation analysis revealed candidate-direct-target genes of *Nr2e1*

Gene name	No. of NR2E1 binding sites	No. of SOX9 binding sites
*Lhx2*	35	13
*Ppp1r9a*	30	2
*Gap43*	29	3
*Myh10*	23	2
*Fezf2*	20	3
*Cux1*	19	9
*Ppp1r9b*	19	1
*Epha4*	18	4
*Nr2e1*	17	4
*Slc1a3*	17	1
*Atrx*	15	2
*Neurod6*	14	4
*Rgma*	14	3
*Mtap1b*	13	5
*Kif5c*	13	3
*Bzw2*	13	2
*Cntn2*	12	1
*Tbr1*	11	1
*Dpysl2*	10	2
*Msx1*	9	3
*Efnb2*	9	2
*Neurod1*	9	2
*Rufy3*	9	2
*Apc*	9	1
*Notch1*	8	3
*Hes6*	8	2
*Racgap1*	8	2
*Edg1 (S1pr1)*	7	1
*Id3*	6	3
*Otx1*	6	3
*Elavl3*	6	1
*Pfn1*	5	1
*Neurog2*	4	1
*Sema4g*	4	1
*Tubb3*	3	2
*Dner*	3	1
*Mtpn*	3	1
*Sept4*	3	1
*Hes5*	2	2
*Sox11*	2	1

### Differential expression of the transcription factor *Lhx2* validated our LongSAGE results

**Fig. 7:**
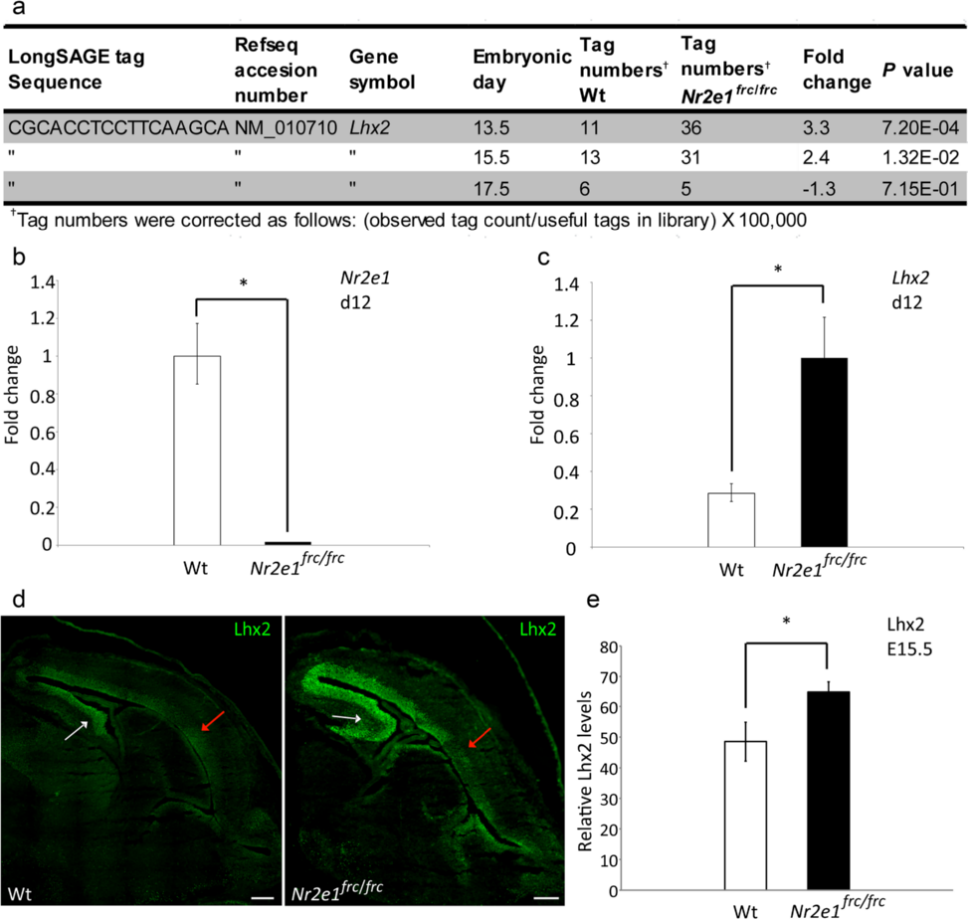
Validation of the differential abundance of *Lhx2* LongSAGE tags in Wt vs. *Nr2e1* ^*frc*/*frc*^ embryos. **a** The tag count results, mapping to *Lhx2* at the three different embryonic days, are presented. Columns one to three present the tag sequence, accession number, and gene symbol corresponding to *Lhx2*; columns four to six, the corrected tag numbers recovered from DiscoverySpace in both Wt and *Nr2e1* ^*frc*/*frc*^ embryos at each time point (E13.5, 15.5, and 17.5); column seven, the fold change between the tag numbers corresponding to *Lhx2* found in Wt and *Nr2e1* ^*frc*/*frc*^ embryos at each time point; and column eight, the associated *P* values obtained using the Audic-Claverie statistical method. According to this approach, *Lhx2* expression level is significantly upregulated at both E13.5 and E15.5 in *Nr2e1* ^*frc*/*frc*^ embryos. **b** Wt embryonic stem cells (ESC) submitted to a neurogenic differentiation protocol demonstrated expression of *Nr2e1* at 12 days of differentiation (d12) whereas, as expected, *Nr2e1* ^*frc*/*frc*^ ESC did not express *Nr2e1* (*, *P* < 0.001). **c** Quantitative RT-PCR reveals that the *Lhx2* mRNA level is upregulated by ~3.6 fold in *Nr2e1* ^*frc*/*frc*^ ESC at d12 compared to Wt ESC (*, *P* < 0.01). **d** Immunofluorescence using an anti-Lhx2 antibody (green) demonstrated a similar expression pattern for the Lhx2 protein along the ventricular/subventricular zone (VZ/SVZ) of the lateral telencephalon in E15.5 *Nr2e1* ^*frc*/*frc*^ embryos compared to Wt. White arrows, medial pallium; red arrows, dorsal pallium; scale bar, 200 μm. **e** Lhx2 protein level was increased by ~1.3 fold along the VZ/SVZ of the lateral telencephalon in E15.5 *Nr2e1* ^*frc*/*frc*^ embryos compared to Wt (*, *P* < 0.05). (**c**), (**d**), (**e**) Sample Student’s *t*-test were performed, N = 3; error bars, standard error of the mean

To expand our understanding of the relationship between *Nr2e1* and *Lhx2*, and simultaneously further validate the results obtained from the LongSAGE tag libraries, we undertook two biological assays; one each *in vitro* and *in vivo*. First we retrieved with DiscoverySpace 4.0 the LongSAGE tag sequence mapping to *Lhx2*, and the corrected number of tags from each library (Fig. 7a). This showed that *Lhx2* levels were significantly increased in *Nr2e1*^*frc*/*frc*^ libraries at two different time points (E13.5, and E15.5).

For *in vitro* studies, we chose a method of neurogenesis from adherent-monoculture of ESC, which sequentially mimics the development of cortical neurons over the course of 21 days of differentiation [74]. In this system: neural induction starts at day 0 (d0) of differentiation; neurogenesis starts at d6 with the generation of subplate neurons and deep layer neurons between d7 and d9, followed by upper cortical neurons around d12; finally there is a wave of gliogenesis by d21 [75]. The formation of subplate neurons corresponds to *in vivo* E10.5-E13.5, deep layer neurons to E11.4-E14.5, and upper cortical neurons to E13.5-E16.5 [75]. Hence, this culture system encompass the key time periods for the function of *Nr2e1* in brain development. Using this method, we first detected *Nr2e1* expression at d6, which then increased and peaked at d12 (data not shown). Further investigation using qRT-PCR at this latter time point not only showed a significant difference between Wt and *Nr2e1*^*frc*/*frc*^ cells for the *Nr2e1* gene (Fig. 7b), but also demonstrated a significant increase in expression in *Nr2e1*^*frc*/*frc*^ cells when compared to Wt cells for the *Lhx2* gene (*P* < 0.01) (Fig. 7c) [75]. This result was consistent with a model of *Lhx2* being a direct target of, and repressed by, Nr2e1.

For *in vivo* studies, we examined the expression pattern of the Lhx2 protein by immunofluorescence in Wt and *Nr2e1*^*frc*/*frc*^ E15.5 embryos. The results showed similar Lhx2 protein localization when comparing Wt and *Nr2e1*^*frc*/*frc*^ embryos; along the VZ/SVZ of the developing forebrain. Furthermore, for both Wt and *Nr2e1*^*frc*/*frc*^, expression levels varied from high in the medial pallium to low in the dorsal pallium (Fig. 7d). However, relative quantification of Lhx2 between Wt and *Nr2e1*^*frc*/*frc*^, along the VZ/SVZ of the dorsal-lateral telencephalon, revealed a significant increase of Lhx2 protein in the *Nr2e1*^*frc*/*frc*^ embryos when compared to Wt (*P* < 0.01) (Fig. 7e). Thus, the significant increase at the mRNA level for the *Lhx2* gene results in a significant increase at the protein level along the VZ/SVZ of the dorsal-lateral telencephalon at E15.5. These data add further support to the hypothesis that *Nr2e1* directly-negatively regulates *Lhx2* expression in the dorsal-lateral telencephalon during development.

## Conclusions

In this work, we have generated a list of 1279 genes that are differentially expressed in response to altered *Nr2e1* expression during *in vivo* neocortex development; this list was a critical part of our own studies, but is also an important resource for others (Additional file 1: Table and Additional file 2: Table S2). To create this list, we profiled the transcriptomes of Wt and *Nr2e1*^*frc*/*frc*^ embryos by generating LongSAGE libraries through LCM of the VZ/SVZ from the dorsal-lateral telencephalon. To further focus the work on the role of *Nr2e1* during neocortex development, we chose two time points that spanned the early to mid-neurogenic stages (E13.5, E15.5), and one time point corresponding to the early switch from neurogenesis to gliogenesis (E17.5). Thus, from six LongSAGE libraries we identified 1279 candidate genes comprising both direct and indirect targets of *Nr2e1* during neocortex development. This list can now be mined by us, and many other groups for the anticipated numerous co-suppressors, co-activators, and direct targets making up the molecular mechanisms of the nuclear-receptor transcription-factor *Nr2e1*.

We have further refined this list of 1279 differentially expressed genes, culminating in a focused list of 64 candidate direct-targets of NR2E1 binding during nervous system development, for our own studies and as a resource for others (Additional file 2: Table S2). This was accomplished by performing two sequential analyses; 1) a TFBSs prediction analysis, using oPOSSUM, to identify novel direct targets of *Nr2e1,* and 2) a GO term overrepresentation analysis, to extract biological meaning from the latter generated list. This procedure included the generation of a novel NR2E1 PWM based on available information from the literature (Additional file 3: Table S3); the derived matrix and logo are provided (Fig. 4a). We used this matrix, in combination with the LongSAGE results, in a bioinformatic experiment to identify novel direct-target genes of *Nr2e1*. The resulting list of GO terms coming from this analysis (Table 1) contained genes differentially expressed, and predicted to contain NR2E1 binding sites within their promoter regions (Additional file 2: Table S2). The GO term category “nervous system development” contained 64 such genes (Fig. 5a, Additional file 2: Table S2); a list that was used in subsequent analyses.

Our approach suggested distinct roles for *Nr2e1* during different neocortex developmental stages. Analyses performed on the differential-tag ratio for the 1279 Refseq accession numbers of differentially-regulated genes retrieved from the Wt and *Nr2e1*^*frc*/.*frc*^ libraries, revealed a positive correlation of the differential abundance at E13.5 and E15.5, whereas a negative correlation was obtained when comparing the two previous time points to E17.5 (Fig. 3). Thus, the differential-tag ratios found at E13.5 and E15.5 were more similar to each other than when compared to E17.5. From E13.5 to E17.5, the neocortex undergoes drastic changes, including the formation of the SVZ, a layer of cells being seeded by the VZ, and a progressive switch from neurogenesis to gliogenesis. Our results indicate that *Nr2e1* has a more similar effect in the early stages of neurogenesis (E13.5 and E15.5) compared to later stages when the switch from neurogenesis to gliogenesis occurs. The mechanism driving these changes may depend on the level of *Nr2e1* expression, which peaks at E13.5 and gradually decreases until birth [18].

The SOX9 transcription factor may be an important co-interactor of NR2E1 in regulating numerous target genes during nervous system development. A co-factor analysis revealed enrichment for binding sites predicted to be bound by SOX9 within the vicinity of the predicted NR2E1 binding sites (Table 2); results that remained significant after calculating empirical *P* values on our list of candidate co-interactors (Additional file 4: Table S4). In addition, the spatial, temporal, and strength of expression of *Sox9* strongly supported a biological relationship with *Nr2e1* [70]. Interestingly, others have shown that *Sox9* may be involved in the acquisition of gliogenic competence of neural stem/progenitor cells during central nervous system (CNS) development [76]. Together these data suggest that co-interaction between the SOX9 transcription factor and NR2E1 may regulate the expression of 40 of the 64 genes involved in nervous system development (Additional file 2: Table S2).

The Sox family of transcription factors may generally be important co-interactors of *Nr2e1* in regulating target genes during nervous system development. This family comprises 20 genes with several members expressed in neural stem/progenitor cells of the CNS, and peripheral nervous system [77–79]. They have been shown to act as either transcriptional activators or repressors by binding to similar (A/T)(A/T)CAA(A/T)G DNA motifs [78]. Recently, one of these family members, Sox2, has been shown to form a regulatory complex with Nr2e1 in adult NSC [64]. Interestingly, our co-factor TFBSs analysis revealed enrichment for the presence of associated binding sites not just for SOX9, but also three additional Sox family members (Sox17, Sox5, and SRY) within the vicinity of predicted NR2E1 binding sites for genes of the “nervous system development” GO term category (Table 2). These additional Sox family members also showed expression overlap with *Nr2e1*, and at least one of these members, Sox5 has been shown to bind to *Fezf2*-conserved-enhancer sequences, resulting in a direct repression of *Fezf2* in neocortex development [80]. LongSAGE tags mapping to *Sox5* were found in our libraries and *Fezf2* was found significantly upregulated in the *Nr2e1*^*frc*/*frc*^ library at E15.5 (7.4 fold, *P* < 0.05, Fig. 5a). Hence our data suggests a specific testable hypothesis by which *Nr2e1* potentially regulates *Fezf2* expression through its interaction via the Sox5 protein in neocortex development. In conclusion, our data supports the hypothesis that generally the Sox family members play an important role as co-interactors of NR2E1.

*Lhx2* may be an important direct-target gene of Nr2e1, with SOX9 as a co-interactor. In *Nr2e1*-null embryos, premature neurogenesis has been reported to occur from E9.5 to E14.5 in both the dorsal and ventral telencephalon [9]. Overexpression of *Lhx2* has been reported to prolong neurogenesis in hippocampal development, resulting in generation of neurons from progenitors that would normally produce astrocytes [72]. Additionally, conditional inactivation *of Lhx2* in neocortical development affects the fate of PC, resulting in a phenotype highly similar to that observed in *Nr2e1*-null embryos; with a reduction in the number of PC populating the VZ and premature neurogenesis in the neocortex of *Lhx2*-null embryos [73]. This latter phenomenon appears to involve the notch signalling pathway, with a downregulation of *Hes1* being observed along the VZ of *Lhx2*-null embryos and aberrant expression of the *Notch* encoding gene along the medial to lateral dorsal telencephalon [73]. Notch pathway genes such as *Notch1*, *Hes5*, and *Hes6* were also found differentially regulated in the *Nr2e1*^*frc*/*frc*^ library when compared to Wt in our LongSAGE analysis (*Notch1*, E13.5, −6.8 fold, *P* < 0.05; *Hes5*, E13.5, −6.8 fold, *P* < 0.01; E17.5, −10.3 fold, *P* < 0.01; *Hes6*, E13.5, 9 fold, *P* < 0.001) (Fig. 5a, Additional file 1: Table S1 and Additional file 2: Table S2). Protein regulatory networks in NSC have been demonstrated to be highly dosage dependent. For instance, phenotypic analyses of Pax6 in gain- or loss-of-function mutant cortices have shown similar phenotypic outcome, with both more or less of the protein resulting in increased neurogenesis throughout development [81]. Hence, akin to *Pax6,* our current results highlight a testable hypothesis in which premature neurogenesis observed in *Nr2e1*^*frc*/*frc*^ embryos [9] could be due to the upregulation of Lhx2 protein along the VZ/SVZ of the dorsal telencephalon; a phenomenon that most likely includes the concerted effect of deregulation of other Notch pathway encoding genes. Our analysis also predicted that the transcription factor pathway regulated by NR2E1 involves interaction with SOX9, which has been shown to be involved in the acquisition of gliogenic competence of neural stem/progenitor cells during CNS development [76]. Hence, our results highlight yet another testable hypothesis for the discovery of a possible novel pathway by which *Nr2e1* regulates neurogenesis, which includes *Lhx2* as one of the direct-target genes, and SOX9 as a co-interactor.

## Methods

### Ethics statement

All procedures involving animals were in accordance with the Canadian Council on Animal Care and UBC Animal Care Committee (Protocol A11-0412).

### LongSAGE libraries generation

Libraries were generated from tissue samples obtained by LCM of dorsal VZ/SVZ from the telencephalon of wild-type (Wt) and *Nr2e1*^*frc*/*frc*^ embryos at E13.5, E15.5, and E17.5 as described by us previously [31]. Briefly, one embryo per genotype at each developmental time point was sectioned at 20 μm thickness to generate the tissue samples. Sections from each embryo underwent LCM, and the isolated tissue was pooled and RNA extracted using an RNeasy Micro Kit (Qiagen Inc., Mississauga, Ontario, Canada). The LongSAGE-lite method was used to construct the libraries using 15 to 86 ng of high quality RNA from each embryo [31, 37, 82]. Each library was sequenced to a depth of >100,000 raw tags and the processed data is accessible on the Mouse Atlas of Gene Expression project website (http://www.mouseatlas.org/) and the NIH SAGEmap data repository http://www.ncbi.nlm.nih.gov/projects/SAGE/) [83].

### LongSAGE data analysis

LongSAGE libraries were analysed using the DiscoverySpace 4.0 application (http://www.bcgsc.ca/platform/bioinfo/software/ds) [44]. The library data were electronically filtered based on procedures previously described by us [45, 84]. Briefly, duplicated ditags (identical copies of a ditag) and singletons (tags counted only once) were retained for analysis. Sequence data were filtered for bad tags (tags with one N-base call), and linker-derived tags (artefact tags). Only tags with a sequence quality factor greater than 99 % were included in the analysis. Sequence tag comparisons between Wt and *Nr2e1*^*frc*/*frc*^ libraries were performed and a *P*-value cutoff < 0.05 using the Audic-Claverie statistical method was used [46]. LongSAGE tags exhibiting differential expression levels were mapped to transcripts in the NCBI Reference Sequence (Refseq) collection (version 52, released March 8^th^ 2012) and Ensembl gene collection (version 66, released February 2012).

### NR2E1 binding site profile construction

No position-weight matrix (PWM) was available in public databases to model NR2E1 transcription factor binding site (TFBS) specificity. Thus, a literature review was conducted and the sequences reported to be bound by NR2E1 were compiled. Next, the MEME motif discovery tool (http://meme-suite.org/) was applied, with default parameter settings, to identify a DNA sequence pattern within the data [85].

### oPOSSUM promoter analysis

A pooled list of RefSeq accession numbers for transcripts exhibiting differential expression between Wt and *Nr2e1*^*frc*/*frc*^ genotypes, at least at one of the three different time points, was subjected to an oPOSSUM TFBS analysis. The oPOSSUM software was run using default settings with both the constructed NR2E1 PWM and the JASPAR core vertebrate PWM collection (http://www.cisreg.ca/oPOSSUM/) [39, 40]. Briefly, for each Refseq accession number, oPOSSUM automatically retrieved the genomic DNA sequences around annotated transcription start sites (TSS) in Ensembl (plus 5000 bp of both upstream and downstream sequence), performed an alignment of the orthologous sequences (human to mouse), and extracted non-coding DNA sequences that are conserved above a predefined threshold (default: top 10 % of conserved regions, minimum conservation 70 %). oPOSSUM results include the positions of predicted TFBSs, and the scores of the sites.

### GO term enrichment analysis

Refseq accession numbers for those genes predicted to contain NR2E1 binding sites in the oPOSSUM database were submitted to the DAVID service (http://david.abcc.ncifcrf.gov/summary.jsp) for GO term annotation enrichment analysis [50, 51]. The Refseq identifiers were converted to DAVID identifiers (IDs), using the DAVID knowledgebase [59]. GO biological-process term enrichment was assessed relative to the entire set of mouse genes as provided by DAVID. Results were filtered to exclude those enriched GO terms associated with 2 or less submitted genes. A significant *P*-value threshold was applied using a multiple testing correction (Bonferroni, *P* value < 0.05).

### Clustering-correlation

Expression clustering was performed on the differential-tag ratios of the initial list of 1279 differentially expressed genes, and the genes annotated within the enriched GO term “nervous system development” category using the Gene Cluster software [47]. Hierarchical clustering was performed on both the gene list and the embryonic stages using Spearman correlation with complete linkage clustering. Tag counts were corrected to account for library sizes; (observed tag counts/total useful tags) X 100,000, and tags having a count value of “0” (no expressed tags) were adjusted to a value of “1” for fold change calculations only. Spearman rank correlation analyses on the differential-tag ratios were performed using STATISTICA 12.0 (Statsoft, Inc., Tulsa, OK, USA). For these latter analyses, genes were considered as valid when their differential-tag ratios between Wt and *Nr2e1*^*frc*/*frc*^ were not equal to zero.

### Co-factor TFBS enrichment analysis and transcription factor candidate evaluation

A customized bioinformatics analysis, based on the oPOSSUM combination site analysis feature, was performed to identify TFBS patterns that were significantly enriched in the vicinity of predicted NR2E1 binding sites for the 64 candidate genes found in the “nervous system development” GO term category. Sites within 100 bp of predicted NR2E1 binding sites were retrieved from the oPOSSUM database. Sites overlapping an NR2E1 motif were excluded. Both the NR2E1 sites and proximal sites were subject to the default oPOSSUM parameters of conservation level (top 10 % of conserved regions with a minimum percentage identity of 70 %), threshold level (default matrix score threshold of 80 %), and search region level (5000 bp upstream and downstream of TSS). The analysis was performed against a background of 500 genes selected randomly from the oPOSSUM database. Over-representation results were considered significant based on a Z score (>10) and a Fisher score (<0.01) according to the literature [40]. To further validate the significance of these results, additional oPOSSUM co-factor analyses were performed on 1000 sets of 64 genes selected randomly from the list of 770 genes enriched for GO terms, using the same analysis parameters and the same set of 500 background genes as the one described above for the “nervous system development” gene set. The significance of the Z and Fisher score for each of the co-factors was determined by empirical *P* value, computed as “n/N” where “n” is the number of times the Z and Fisher score from set of the random trials for the co-factor was more significant than the Z and Fisher score from the “nervous system development” set for that co-factor, divided by the total number “N” of random trials (in this case 1000). These results are shown in Additional file 4: Table S4.

Transcription factors with enriched binding site predictions were additionally assessed for their expression pattern at E13.5, 15.5, and 18.5 using images from the Allen Mouse Brain Atlas (ABA, http://www.brain-map.org/) [69]. Expression results from transcription factors with enriched binding site predictions that were unavailable from the ABA were evaluated using other publicly available resources; Eurexpress (http://www.eurexpress.org/ee/), GenePaint (http://www.genepaint.org/Frameset.html), and the primary literature [86, 87]. The expression pattern was summarized according to the specificity and strength of the expression along the VZ/SVZ, and other forebrain structures. The relevance of the expression pattern for each transcription factor was scored as absent (−), low (+), moderate (++), or high (+++); where absence of VZ/SVZ expression, or ubiquitous expression in the entire embryo forebrain, was scored as +, whereas strong and restricted expression along the VZ/SVZ was scored as +++. The transcription factor having a high score (+++) was retained as the most interesting candidate. For the highest-scoring transcription factors we cross-validated the expression pattern by looking for the number of corresponding tags in the LongSAGE libraries.

### Embryos preparation

Timed-pregnant mice were euthanized by cervical dislocation, and embryos at E15.5 were dissected and fixed in 4 % paraformaldehyde (PFA) with 0.1 M PO buffer (0.1 M Na_2_HPO_4_, pH 8.0) for 6 h at 4 °C. The embryos were then cryoprotected as described in the literature, and embedded in optimal cutting temperature (OCT) compound (Tissue-tek, Torrance, California, USA) on dry ice [11]. Embryos were sectioned at 20 μm using a Cryo Star HM550 cryostat (MICROM International, Kalamazoo, Michigan, USA), and mounted for immunofluorescence.

### Immunofluorescence and imaging analysis

For antibody staining, 20 μm sagittal cryosections from embryos were rehydrated in sequential washes of 1x phosphate buffered saline (PBS), permeabilized in PBS with 0.3 % triton, and blocked with 1 % BSA (bovine serum albumin) in PBS triton 0.3 % for 1 h at room temperature. Goat anti-Lhx2 primary antibody (1:1000) (Santa Cruz, Dallas, TX, USA, sc-19344) was incubated overnight at 4 °C. Rabbit anti-goat Alexa 488 (1:1000) (Invitrogen, Burlington, Ontario, Canada, A21222) was incubated for 2 h at room temperature in the dark. Tiled images were retrieved with an Olympus BX61 motorized fluorescence microscope at 20X magnification (Olympus America Inc., Center Valley, Pennsylvania, USA). Intensity quantification was performed using Image-Pro (Media Cybernetics Inc., Bethesda, Maryland, USA). The relative intensity level of Lhx2 was calculated as described in the literature [88]. Briefly, the sum of the signal intensity was divided by the area selected and multiplied by the thickness of the section and the number of sections. A background correction was applied using the signal intensity resulting from Hoechst staining for each sections quantified. A total of 28 different sections were assessed on six embryos, three different animals for each genotype (Wt and *Nr2e1*^*frc*/*frc*^). All values represent the mean ± standard error of the mean (SEM). Statistical analysis was performed using Student’s *t*-test.

### Embryonic stem cells culture

ESC from Wt and *Nr2e1*^*frc*/*frc*^ blastocysts were derived, and maintained in culture as described in the literature [89]. The two cell lines used were mEMS1239 (B6129F1-*Nr2e1*^*frc*/*frc*^, *Hprt1*^*b-m3*^/Y), and mEMS1271 (B6129F1-*Nr2e1*^+/+^, *Hprt1*^*b-m3*^/Y).

The ESC differentiation procedure involved the use of an adapted method of neurogenesis from adherent monoculture [74, 75]. Briefly, the cells were seeded at low density (~10,000 cells/mm^2^) on gelatin coated dishes, in a chemically defined medium exempt of cyclopamine, and maintained in culture for 12 days (fresh media every two days). RNA aliquots were prepared on day 12 and were used for quantitative RT-PCR (qRT-PCR).

### Quantitative RT-PCR

RNA from ESC grown in an adapted method of neurogenesis from adherent monoculture, collected on day 12, was extracted using Qiagen RNA Mini Plus kit (Qiagen Inc., Mississauga, Ontario, Canada). RNA was treated with Qiagen DNase kit (Qiagen Inc., Mississauga, Ontario, Canada), and cDNA was generated using Superscript III Master Mix kit (Invitrogen, Burlington, Ontario, Canada). cDNA quantification was performed using ABI Taqman® assays specifically designed for *Nr2e1* (Mm00455855_m1), and *Lhx2* (Mm00839783_m1) (Applied Biosystems Inc., Foster city, USA). The 7500 fast real-time PCR system and Taqman® fast universal PCR Master Mix were used (Applied Biosystems Inc., Foster city, USA). The cycle threshold (Ct) value was defined as the number of cycles required for the fluorescent signal to cross a threshold above the background signal, and is inversely proportional to the amount of target cDNA. All values represent the mean ± SEM. Statistical analysis was performed using Student’s *t*-test.

### Availability of supporting data

LongSAGE processed data is accessible on the Mouse Atlas of Gene Expression project website (http://www.mouseatlas.org/) and the NIH SAGEmap data repository (http://www.ncbi.nlm.nih.gov/projects/SAGE/). All additional supporting data is included as additional files.

## Additional files

Additional file 1: Table S1. List of the 1387 differentially abundant tag sequences mapping to the 1279 genes, differentially expressed in response to altered *Nr2e1* levels during *in vivo* neocortex development and the corresponding tag numbers, calculated fold changes and *P* values at E13.5, E15.5, and E17.5. Columns one and two present the gene ID/symbol, associated tag sequence(s) and corresponding Refseq ID for each gene. Columns three and four, seven and eight, eleven and twelve present the number of tags found in Wt and *Nr2e1* ^*frc*/*frc*^ libraries at E13.5, E15.5, and E17.5 inclusively. Column five, nine, and thirteen present the fold change values resulting from calculations based on the tag number values found in the previous columns (details, see Methods). Column six, ten, and fourteen present the associated *P* values calculated using the Audic-Claverie statistical test for each corresponding tags at the three different time points (E13.5, E15.5 and E17.5). Additional file 2: Table S2. List of the 1279 genes, differentially expressed in response to altered *Nr2e1* levels during *in vivo* neocortex development, and the summary of significant findings for each. Columns one and two present the gene ID/symbol and associated transcript ID for each gene. Columns three to five present the genes having significantly differentially abundant tags at E13.5, E15.5, and E17.5, respectively. Columns six to nine present the genes retained after performing subsequent bioinformatics analyses. *, Multiple tags that were either up- or down-regulated but mapped to the same gene; details of the tag sequences and the direction of change are depicted. NA, not applicable. Additional file 3: Table S3.
*Nr2e1* binding sites containing sequences extracted from the literature and used to generate a position weight matrix. Columns one and two present the gene names and species origin of the DNA sequence analyzed. Column three presents the DNA sequence analyzed. Column four presents the PAZAR ID matching the DNA sequence presented in column three. Column five presents the PubMed ID associated with the manuscript from which the sequences were derived. NA, not applicable. Additional file 4: Table S4. Empirical *P* values extracted from the Z-scores and Fisher scores of each candidate-NR2E1 co-interactors. Column one presents the transcription factor symbol. Column two and three present the calculated empirical *P* values for both the Z-score and Fisher score of the corresponding transcription factor.

## Abbreviations

ABA: Allen Mouse Brain Atlas
BSA: Bovine Serum Albumin
CNS: Central Nervous System
ESC: Embryonic Stem Cells
GO: terms Gene Ontology terms
LCM: Laser Capture Microdissection
NSC: Neural Stem Cells
OCT: Optimal Cutting Temperature
PBS: Phosphate Buffered Saline
PC: Progenitor Cells
PFA: Paraformaldehyde
PO: Na_2_HPO_4_, pH 8.0
PWM: Position Weight Matrix
TFBS: Transcription Factor Binding Site
SAGE: Serial Analysis of Gene Expression
SELEX: Systematic Evolution of Ligands by Exponential Enrichment
TSS: Transcription Start Sites
SVZ: Subventricular Zone
UCSC: University of California, Santa Cruz
UTR: Untranslated Region
VZ: Ventricular Zone
Wt: Wild-Type
